# Complete Genome Sequence of Petrimonas sp. Strain IBARAKI, Assembled from the Metagenome Data of a Culture Containing Dehalococcoides spp.

**DOI:** 10.1128/genomeA.00384-18

**Published:** 2018-05-03

**Authors:** Kentaro Ikegami, Yuto Aita, Akino Shiroma, Makiko Shimoji, Hinako Tamotsu, Noriko Ashimine, Misuzu Shinzato, Shun Ohki, Kazuma Nakano, Kuniko Teruya, Kazuhito Satou, Takashi Hirano, Masafumi Yohda

**Affiliations:** a Department of Biotechnology and Life Science, Tokyo University of Agriculture and Technology, Koganei, Tokyo, Japan; b Okinawa Institute of Advanced Sciences, Uruma, Okinawa, Japan

## Abstract

The complete genome sequence of Petrimonas sp. strain IBARAKI in a Dehalococcoides-containing culture was determined using the PacBio RS II platform. The genome is a single circular chromosome of 3,693,233 nucleotides (nt), with a GC content of 44%. This is the first genome sequence of a Petrimonas species.

## GENOME ANNOUNCEMENT

Previously, we tried to obtain bacteria that support the growth of Dehalococcoides spp. from the consortium containing Dehalococcoides mccartyi IBARAKI (1). We obtained single colonies after several rounds of shake agar cultures using the medium for Desulfovibrio species. Then, we supplemented the clone in the culture of the isolate D. mccartyi UCH-ATV1 (2). As expected, the mixed consortium exhibited significantly high dechlorination activity for trichloroethene. By denaturing gradient gel electrophoresis (DGGE) analysis for 16S rRNA and metagenome analyses using next-generation sequencers, it was revealed that the constructed consortium consists of D. mccartyi UCH-ATV1, Desulfovibrio sp., Petrimonas sp., and Clostridium spp. Then, we carried out whole-metagenome sequencing using the PacBio RS II platform. The metagenomic DNA was extracted from the consortium and converted into 20-kb template libraries without shearing. Size selection was not applied. We used 30 single-molecule real-time (SMRT) cells for two sequencing runs with P6-C4 chemistry and 240-min movies. In the first run using 16 cells, 1.4 million reads with an average length of 4.2 kb were obtained. In the second run using 14 cells, 533,000 reads with an average length of 3.9 kb were obtained. We applied HGAP version 2 with a 6-kb minimum seed read length, 30× target coverage, and a 1.4-Mb genome size, and HGAP version 3 with the default minimum seed read length, 25× target coverage, and a 1.4-Mb genome size to each of the sequencing data. A total of 485 contigs were assembled. We attempted to overlap and join those contigs using Minimus2, and a circular sequence was constructed. We polished the circular sequence using Quiver until no variants were detected. The length of the complete genome sequence is 3,693,233 nucleotides (nt). The number of coding sequences (CDSs) was 2,992. In addition, two pairs of rRNA genes (5S rRNA, 16S rRNA, and 23S rRNA) and 48 tRNA genes were identified. The 16S rRNA gene showed an identity of 99% (1,403/1,407) with that of Petrimonas sulfuriphila strain BN3^T^ (GenBank accession number NR_042987) (3). Thus, we confirmed that the genome is that of Petrimonas sp. strain IBARAKI. Petrimonas sulfuriphila strain BN3^T^ was isolated from a producing well of a biodegraded oil reservoir in Canada. The strain is a member of the phylum Bacteroidetes, distantly related to the genera Bacteroides and Tannerella. This organism possesses phenotypic, chemotaxonomic, and phylogenetic traits that do not allow its classiﬁcation as a member of any previously described genus; therefore, it was proposed that this isolate should be described as a member of a novel species of a new genus, Petrimonas. P. sulfuriphila BN3^T^ is a strictly anaerobic chemo-organotrophic organism. Elemental sulfur stimulated the growth of P. sulfuriphila BN3^T^ and is reduced to H_2_S. Similar to P. sulfuriphila BN3^T^, Petrimonas sp. IBARAKI was obtained in the black colonies with sulfide. This is the first genome sequence of a Petrimonas species.

### Accession number(s).

The complete genome sequence of Petrimonas sp. IBARAKI has been deposited in DDBJ/ENA/GenBank under the accession number AP018040.

## References

[1] Yohda M, Yagi O, Takechi A, Kitajima M, Matsuda H, Miyamura N, Aizawa T, Nakajima M, Sunairi M, Daiba A, Miyajima T, Teruya M, Teruya K, Shiroma A, Shimoji M, Tamotsu H, Juan A, Nakano K, Aoyama M, Terabayashi Y, Satou K, Hirano T 2015 Genome sequence determination and metagenomic characterization of a *Dehalococcoides* mixed culture grown on *cis*-1,2-dichloroethene. J Biosci Bioeng 120:69–77. doi:10.1016/j.jbiosc.2014.12.001. 25579666

[2] Yohda M, Ikegami K, Aita Y, Kitajima M, Takechi A, Iwamoto M, Fukuda T, Tamura N, Shibasaki J, Koike S, Komatsu D, Miyagi S, Nishimura M, Uchino Y, Shiroma A, Shimoji M, Tamotsu H, Ashimine N, Shinzato M, Ohki S, Nakano K, Teruya K, Satou K, Hirano T, Yagi O 2017 Isolation and genomic characterization of a *Dehalococcoides* strain suggests genomic rearrangement during culture. Sci Rep 7:2230. doi:10.1038/s41598-017-02381-0. 28533514 PMC5440377

[3] Grabowski A, Tindall BJ, Bardin V, Blanchet D, Jeanthon C 2005 *Petrimonas sulfuriphila* gen. nov., sp. nov., a mesophilic fermentative bacterium isolated from a biodegraded oil reservoir. Int J Syst Evol Microbiol 55:1113–1121. doi:10.1099/ijs.0.63426-0. 15879242

